# Association of LDL-C/HDL-C Ratio With Stroke Outcomes Within 1 Year After Onset: A Hospital-Based Follow-Up Study

**DOI:** 10.3389/fneur.2020.00408

**Published:** 2020-05-15

**Authors:** Li Liu, Ping Yin, Chong Lu, Jingxin Li, Zhaoxia Zang, Yongdan Liu, Shuang Liu, Yafen Wei

**Affiliations:** ^1^Department of Neurology, Heilongjiang Provincial Hospital, Harbin, China; ^2^Department of Neurology, Heilongjiang Provincial Hospital Affiliated to Harbin Institute of Technology, Harbin, China

**Keywords:** stroke, outcomes, lipids, LDL-C/HDL-C ratio, risk factor, epidemiology

## Abstract

Stroke remains a leading cause of death and disability. The low-density lipoprotein cholesterol to high-density lipoprotein cholesterol (LDL-C/HDL-C ratio) ratio has been confirmed to be a predictor of stroke. However, few studies have assessed the prognostic impact of the LDL-C/HDL-C ratio for stroke patients. We aimed to investigate the relationship between the LDL-C/HDL-C ratio and the prognosis following stroke in Chinese patients. A total of 3,410 patients who had experienced their first ischemic stroke was recruited to this study within 72 h of stroke onset. The patients were followed for at least 12 months. A multivariate regression analysis was used to assess the association between the LDL-C/HDL-C ratio and prognosis following stroke. We considered the LDL-C/HDL-C ratio as a continuous variable and stratified patients according to the LDL-C/HDL-C ratio quartile. A higher LDL-C/HDL-C ratio was associated with lower rates of death, recurrence, and moderate disability (defined as a modified Rankin scale score >2) at 3 months. Using group 1 as the reference group, the relative risk (RRs) at 3 months for death were 0.45 (95% confidence interval [CI]: 0.27, 0.77) for group 2, 0.58 (95% CI: 0.34, 0.98) for group 3, and 0.97 (95% CI: 0.60, 1.56) for group 4; for recurrence, the RRs were 0.75 (95% CI: 0.56, 0.99) for group 2, 0.65 (95% CI: 0.48, 0.89) for group 3, and 0.55 (95% CI: 0.39, 0.78) for group 4; and for moderate disability, the RRs were 0.74 (95% CI: 0.55, 0.99) for group 2, 0.65 (95% CI: 0.47, 0.89) for group 3, and 0.55 (95% CI: 0.39, 0.77) for group 4. At 12 months, patients in group 2 were the most protected against ischemic stroke death (RR: 0.57; 95% CI: 0.34, 0.95). However, there were no associations between the LDL-C/HDL-C ratio and stroke recurrence or moderate disability. A higher LDL-C/HDL-C ratio was found to protect against death, recurrence, and moderate disability at 3 months. However, there was no significant association between the LDL-C/HDL-C ratio and stroke recurrence or moderate disability at 12 months. These results nonetheless suggest that a higher LDL-C/HDL-C ratio was associated with short-term stroke prognosis.

## Introduction

Over the past few decades, stroke has been a leading cause of death and long-term disability, accounting for more than 4% of direct medical expenditures in developed countries (1–3). In China, stroke was the leading cause of death and disability from 1990 to 2017 (4), and almost one-third of the total number of deaths attributable to stroke worldwide occurred in China (5, 6). The economic burden of stroke in China is as high as ¥52 billion per year and mostly comprises long-term post-stroke treatment costs (4, 7). With the increase in life expectancy, disability caused by stroke has seriously hindered social mobility, economic progress, and improvement of living standards for older adults (8). Therefore, it is vital to determine the risk factors that affect prognosis following stroke and to implement treatments to reduce the incidence of new ischemic events.

Increasing evidence points to the low-density lipoprotein cholesterol/high-density lipoprotein cholesterol (LDL-C/HDL-C ratio) ratio as a novel indicator of the risks of both atherosclerotic cardiovascular and cerebrovascular diseases, as it simultaneously takes into account both LDL-C and HDL-C levels (9, 10). A high LDL-C/HDL-C ratio is associated with cardiovascular events (11, 12). Meanwhile, low levels of HDL-C also have a significant impact on prognosis following cerebrovascular diseases (13–16). However, few studies have described the relationship between stroke prognosis and the LDL-C/HDL-C ratio, especially in China. Thus, we aimed to assess the association between the LDL-C/HDL-C ratio and the 12-month prognosis following acute ischemic stroke in China.

## Methods

### Study Design and Study Population

This study recruited consecutive stroke patients who were admitted to the Stroke Unit at Heilongjiang Provincial Hospital for the first time within 72 h of stroke onset between January 2009 and December 2015. A clinical diagnosis of stroke was made according to the World Health Organization's standards and confirmed by neuroimaging (computed tomography/magnetic resonance imaging). Patients who experienced transient ischemic attack and/or hemorrhagic stroke were excluded from this study. TIA was defined as a brief episode of neurological dysfunction resulting from focal cerebral or retinal ischemia, with clinical symptoms typically lasting less than 1 hour and without evidence of acute infarction on imaging (17). The blood lipid levels used in our analysis were measured at the time of admission and at the time of follow-up.

Patients who experienced atherothrombotic infarction were classified according to quartiles of LDL-C/HDL-C ratio and subsequently analyzed by group. All patients were treated using statins and followed for at least 3 months after acute ischemic stroke. At 3 months after the onset of stroke, the subjects' compliance with the use of statins was 100%, and the types of statins used were rosuvastatin (10 mg per day) and atorvastatin (20 mg per day).

This study was approved by the Medical Research Ethics Committee of Heilongjiang Provincial Hospital, and written informed consent was obtained from each participant during recruitment.

### Data Collection and Follow-Up

Standardized questionnaires were used to obtain detailed information from patients at admission, including information on ischemic stroke subtype, neurological deficit, stroke severity, medical history, and laboratory test results. Blood lipid concentrations were measured using a fasting blood sample with established methods and the national unified instrument at Heilongjiang Provincial Hospital medical testing center, which is a nationally certified medical testing center. Follow-up visits were scheduled starting from the date of discharge from the index hospitalization. Outcomes (i.e., mortality, recurrence, and dependency) at 3 and 12 months after stroke were recorded.

Short-term (at 3 months) and long-term (at 12 months) stroke outcomes were determined based on mortality, recurrence, and moderate disability (which was defined as a modified Rankin Scale [mRS] score >2). Death was defined as cumulative all-cause death at the corresponding time point after stroke; information on death was collected from death registries at hospitals or telephone interviews with the patients' family members. Recurrence was defined as any new vascular event (i.e., stroke, myocardial infarction, peripheral vascular events) occurring at least 30 days after the initial stroke onset. Data were collected by face-to-face or telephone interviews by trained neurologists.

The National Institutes of Health Stroke Scale (NIHSS) score at admission and data on the patient's history of hypertension, diabetes, obesity, smoking, or alcohol consumption were reviewed. The severity of stroke was classified into three groups according to the NIHSS: mild (NIHSS score <7), moderate (NIHSS score 8–16), and severe (NIHSS score ≥17).

### Statistical Analysis

We considered the LDL-C/HDL-C ratio to be a continuous variable and stratified patients according to LDL-C/HDL-C ratio quartile as follows: group 1, LDL-C/HDL-C ratio, <2.23; group 2, 2.23–2.87; group 3, 2.88–3.55; and group 4, ≥3.56. We defined an HDL-C concentration of <1.04 mmol/L as low HDL-C and an LDL-C concentration of ≥4.14 mmol/L as high LDL-C to provide a basis for evaluating the impact of the LDL-C/HDL-C ratio on prognosis.

Continuous variables are expressed as means ± standard deviations and categorical variables as percentages. Analysis of variance and the chi-squared test were used to analyze continuous and categorical variables, respectively. We performed a logistic regression analysis to assess the association between the LDL-C/HDL-C ratio and stroke outcomes within 12 months of stroke onset. The three aforementioned stroke outcomes (i.e., mortality, recurrence, and moderate disability) were considered as the dependent variables, and the LDL-C/HDL-C ratio quartile and other covariables (including age; sex; NIHSS score at admission; and history of hypertension, diabetes, obesity, smoking, and/or alcohol consumption) were considered to be independent variables. All statistical analyses were performed using the SPSS 19.0 statistical package (IBM SPSS Statistics for Windows, Version 19.0. IBM Corp., Armonk, NY). All significance tests were two-sided, and *P* values <0.05 were considered significant.

## Results

### Patient Characteristics

**Figure 1:**
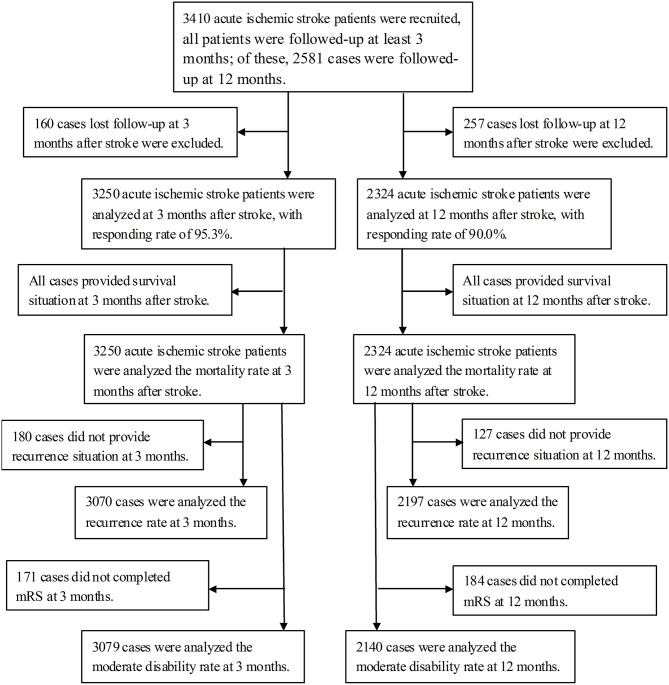
Flow chat of patients' selection.

The study included 3,410 patients with a first-ever diagnosis of stroke, of whom 2,284 (67.0%) were men and 1,126 (33.0%) were women. The mean age of the women was significantly higher than that of the men (66.42 vs. 62.39 years). Most men (47.0%) were aged 50 to 64 years, whereas most women (44.6%) were aged 65 to 79 years. In terms of NIHSS score, there were 2375 (69.7%) patients in the <7 group, 759 (22.3%) in the 7–16 group, and 273 (8.0%) in the ≥17 group. The prevalences of atrial fibrillation, diabetes mellitus, hypertension, obesity, current smoking, and alcohol consumption were 7.3, 31.8, 73.9, 13.4, 39.4, and 20.1%, respectively. The mean LDL-C/HDL-C ratio and concentrations of total cholesterol (TC), triglycerides (TG), HDL-C, LDL-C, and fasting plasma glucose (FPG) were 2.87, 4.96, 1.64, 1.08, 2.96, and 6.91 mmol/L, respectively (Table 1). Flow chat of patients' selection was showed in Figure 1.

**Table 1:** Clinical and demographical characteristics of ischemic stroke patients in men and women.

**Features**	**Total**	**Men**	**Women**	***P***
Cases, *n* (%)	3410	2284 (67.0)	1126 (33.0)	
Age, means ± SD, year	63.72 ± 11.33	62.39 ± 11.41	66.42 ± 10.69	<0.001
Age groups, *n* (%):				<0.001
<50 years	359 (10.5)	288 (12.6)	71 (6.3)	
50~64 years	1495 (43.8)	1073 (47.0)	422 (37.5)	
65~79 years	1250 (36.7)	748 (32.7)	502 (44.6)	
≥80 years	306 (9.0)	175 (7.7)	131 (11.6)	
NIHSS, *n* (%):				<0.001
<7	2375 (69.7)	1641 (71.9)	734 (65.3)	
7~	759 (22.3)	480 (21.0)	279 (24.8)	
≥ 17	273 (8.0)	162 (7.1)	111 (9.9)	
Hypertension, *n* (%):				<0.001
Yes	2521 (73.9)	1619 (70.9)	902 (80.1)	
No	889 (26.1)	665 (29.1)	224 (19.9)	
Diabetes mellitus, *n* (%):				<0.001
Yes	1084 (31.8)	670 (29.3)	712 (36.8)	
No	2326 (68.2)	1614 (70.7)	414 (63.2)	
Atrial fibrillation, *n* (%):				<0.001
Yes	250 (7.3)	137 (6.0)	113 (7.3)	
No	3160 (92.7)	2147 (94.0)	1013 (92.7)	
Obesity, *n* (%):				<0.001
Yes	458 (13.4)	213 (9.3)	245 (21.8)	
No	2952 (86.6)	2071 (90.7)	881 (78.2)	
Smoking, *n* (%):				<0.001
Yes	1342 (39.4)	1193 (52.2)	149 (13.2)	
No	2068 (60.6)	1091 (47.8)	977 (86.8)	
Drinking, *n* (%):				<0.001
Yes	686 (20.1)	670 (29.3)	16 (1.4)	
No	2724 (79.9)	1614 (70.7)	1110 (98.6)	
**LABORATORY ITEMS, MEANS ± SD, MMOL/L**				
TC	4.96 ± 1.11	4.79 ± 1.04	5.32 ± 1.60	<0.001
TG	1.64 ± 1.08	1.61 ± 1.07	1.70 ± 1.12	0.040
HDL-C	1.08 ± 0.29	1.05 ± 0.28	1.16 ± 0.30	<0.001
LDL-C	2.96 ± 0.87	2.89 ± 0.83	3.16 ± 0.92	<0.001
LDL/HDL	2.87 ± 1.01	2.87 ± 0.99	2.86 ± 1.05	0.700
FPB	6.91 ± 2.99	6.78 ± 2.94	7.12 ± 3.07	0.001

### Ischemic Stroke Outcomes Categorized by the LDL-C/HDL-C Ratio

As the LDL-C/HDL-C ratio increased, the 3-month recurrence rate and proportion of patients with moderate disability decreased (*P* < 0.001). However, there was no significant association between mortality at 3 months and the LDL-C/HDL-C ratio (*P* = 0.215). Similarly, there was no significant association between prognosis and the LDL-C/HDL-C ratio at 12 months (*P* = 0.304; Table 2).

**Table 2:** The ischemic stroke outcomes categorized by LDL-C/HDL-Cratio.

**Clinical prognosis**	**Death**	**Recurrence**	**Moderate disability**
3 months:			
<2.23	65 (6.0)	147 (14.3)	148 (14.4)
2.23 ~	22 (2.9)	83 (11.1)	83 (11.1)
2.88 ~	23 (3.2)	66 (9.6)	66 (9.6)
≥3.56	33 (5.1)	51 (2.1)	51 (8.3)
Total	143 (4.4)	347 (11.3)	348 (11.3)
*P*	0.215	<0.001	<0.001
12 months:			
<2.23	68 (8.9)	236 (33.1)	225 (32.4)
2.23 ~	28 (5.3)	157 (30.8)	148 (29.6)
2.88 ~	30 (5.8)	163 (32.5)	149 (30.6)
≥3.56	46 (9.1)	145 (30.5)	133 (29.0)
Total	172 (7.4)	701 (31.9)	655 (30.6)
*P*	0.841	0.449	0.256

### Association Between Outcomes Within 12 Months and the LDL-C/HDL-C Ratio

After adjustment for covariates, a higher LDL-C/HDL-C ratio was associated with better outcomes (death, recurrence, and moderate disability) at 3 months following stroke. When compared to group 1 as the reference group, the RRs at 3 months for death were 0.45 (95% confidence interval [CI]: 0.27, 0.77) for group 2, 0.58 (95% CI: 0.34, 0.98) for group 3, and 0.97 (95% CI: 0.60, 1.56) for group 4; the RRs for recurrence were 0.75 (95% CI: 0.56, 0.99) for group 2, 0.65 (95% CI: 0.48, 0.89) for group 3, and 0.55 (95% CI: 0.39, 0.78) for group 4; and the RRs for moderate disability were 0.74 (95% CI: 0.55, 0.99) for group 2, 0.65 (95% CI: 0.47, 0.89) for group 3, and 0.55 (95% CI: 0.39, 0.77) for group 4. At the 12-month follow-up, the corresponding RRs for mortality were 0.57 (95% CI: 0.34, 0.95) for group 2, 0.73 (95% CI: 0.44, 1.22) for group 3, and 1.23 (95% CI: 0.78, 1.95) for group 4; for recurrence were 0.88 (95% CI: 0.69, 1.14) for group 2, 1.00 (95% CI: 0.77, 1.29) for group 3, and 0.88 (95% CI: 0.68, 1.15) for group 4; and for moderate disability were 0.86 (95% CI: 0.66, 1.11) for group 2, 0.95 (95% CI: 0.73, 1.23) for group 3, and 0.86 (95% CI: 0.69, 1.13) for group 4.

At the 3-month follow-up time point, low HDL-C protected against recurrence and moderate disability (RR: 0.78 [95% CI: 0.61, 0.99] and RR: 0.78 [95% CI: 0.61, 0.99], respectively). High LDL-C was a risk factor for 3-month and 12-month mortality (RR: 1.92 [95% CI: 1.06, 3.46] and RR: 1.97 [95% CI: 1.14, 3.39], respectively; Table 3).

**Table 3:** Association of ischemic stroke outcomes with LDL-C/HDL-C ratio, Low-HDL, and High-LDL within 3 and 12 months of stroke onset (RR with 95%CI).

**Clinical prognosis**	**Death**	**Recurrence**	**Moderate disability**
LDL-C/HDL-C			
3 months:			
<2.23	1.00	1.00	1.00
2.23 ~	0.45 (0.27, 0.77)^*^	0.75 (0.56, 0.99)^*^	0.74 (0.55, 0.99)^*^
2.88 ~	0.58 (0.34, 0.98)^*^	0.65 (0.48, 0.89)^*^	0.65 (0.47, 0.89)^*^
≥3.56	0.97 (0.60, 1.56)	0.55 (0.39, 0.78)^*^	0.55 (0.39, 0.77)^*^
12 months:			
<2.23	1.00	1.00	1.00
2.23 ~	0.57 (0.34, 0.95)^*^	0.88 (0.69, 1.14)	0.86 (0.66, 1.11)
2.88 ~	0.73 (0.44, 1.22)	1.00 (0.77, 1.29)	0.95 (0.73, 1.23)
≥3.56	1.23 (0.78, 1.95)	0.88 (0.68, 1.15)	0.86 (0.66, 1.13)
Low-HDL			
3 months:			
No	1.00	1.00	1.00
Yes	1.22 (0.81, 1.84)	0.78 (0.61, 0.99)^*^	0.78 (0.61, 0.99)^*^
12 months:			
No	1.00	1.00	1.00
Yes	1.15 (0.78, 1.70)	1.15 (0.94, 1.40)	1.13 (0.92, 1.39)
High-LDL			
3 months:			
No	1.00	1.00	1.00
Yes	1.92 (1.06, 3.46)^*^	0.97 (0.63, 1.50)	0.97 (0.63, 1.50)
12 months:			
No	1.00	1.00	1.00
Yes	1.97 (1.14, 3.39)^*^	0.90 (0.64, 1.26)	0.90 (0.64,1.27)

* *Presented P < 0.05*.

### Association Between Outcomes Within 12 Months With the LDL-C/HDL-C Ratio by Trial of ORG 10172 in Acute Stroke Treatment (TOAST) Subtype

From the data in Table 4, it is apparent that prognosis following large-artery atherosclerosis (LA) stroke was similar to that following non-classified strokes. At the 3-month follow-up period, the risks of recurrence and moderate disability decreased as the LDL-C/HDL-C ratio increased in patients with LA. The RRs for recurrence and moderate disability were 0.67 (95% CI: 0.48, 0.93) for group 2, 0.62 (95% CI: 0.44, 0.89) for group 3, and 0.42 (95% CI: 0.28, 0.64) for group 4. Meanwhile, participants in group 2 had the lowest RR for ischemic stroke mortality at 3 months (RR: 0.51; 95% CI: 0.29, 0.90). There was no association between LDL-C/HDL-C ratio subgroup with LA mortality at 12 months. Moreover, there was no significant association between cardioembolisms or small-artery occlusion lacunar stroke prognosis and LDL-C/HDL-C ratio group.

**Table 4:** Association of ischemic stroke outcomes with LDL-C/HDL-C ratio within 3 and 12 months of stroke onset by TOAST (RR with 95%CI).

**Clinical prognosis**	**Death**	**Recurrence**	**Moderate disability**
LA			
3 months:			
<2.23	1.00	1.00	1.00
2.23 ~	0.51 (0.29, 0.90)^*^	0.67 (0.48, 0.93)^*^	0.67 (0.48, 0.93)^*^
2.88 ~	0.77 (0.44, 1.37)	0.62 (0.44, 0.89)^*^	0.62 (0.44, 0.89)^*^
≥3.56	1.00 (0.59, 1.73)	0.42 (0.28, 0.64)^*^	0.42 (0.28, 0.64)^*^
12 months:			
<2.23	1.00	1.00	1.00
2.23 ~	0.69 (0.39, 1.23)	0.84 (0.62, 1.23)	0.81 (0.60, 1.10)
2.88 ~	0.95 (0.53, 1.72)	0.98 (0.72, 1.34)	0.93 (0.68, 1.27)
≥3.56	1.27 (0.73, 2.21)	0.84 (0.62, 1.13)	0.93 (0.68, 1.27)
CE			
3 months:			
<2.23	1.00	1.00	1.00
2.23 ~	0.61 (0.10, 3.67)	0.72 (0.17, 3.02)	0.72 (0.17, 3.02)
2.88 ~	0.28 (0.03, 2.84)	0.31 (0.53, 1.84)	0.31 (0.53, 1.84)
≥3.56	3.99 (0.81, 19.69)	1.41 (0.30, 6.63)	1.41 (0.30, 6.63)
12 months:			
<2.23	1.00	1.00	1.00
2.23 ~	0.37 (0.55, 2.48)	0.87 (0.23, 3.31)	0.89 (0.23, 3.45)
2.88 ~	0.20 (0.02, 1.87)	1.19 (0.28, 5.07)	0.91 (0.19, 4.21)
≥3.56	12.10 (1.45, 100.90)	0.67 (0.15, 2.91)	0.35 (0.62, 1.96)
SA			
3 months:			
<2.23	1.00	1.00	1.00
2.23 ~	-	1.08 (0.43, 2.77)	1.08 (0.43, 2.77)
2.88 ~	0.23 (0.03, 2.17)	1.08 (0.43, 2.77)	0.10 (0.41, 2.42)
≥3.56	0.25 (0.03, 1.93)	0.10 (0.41, 2.42)	1.21 (0.50, 2.94)
12 months:			
<2.23	1.00	1.00	1.00
2.23 ~	-	1.32 (0.71, 2.44)	1.32 (0.71, 2.44)
2.88 ~	0.56 (0.13, 2.36)	1.14 (0.64, 2.04)	1.05 (0.58, 1.90)
≥3.56	0.41 (0.08, 2.00)	1.00 (0.54, 1.85)	0.96 (0.52, 1.80)

* *Presented P < 0.05; LA, Large-artery atherosclerosis; CE, cardioembolism; SA, small-artery occlusion lacunar*.

## Discussion

In this study, we reported the relationship between outcomes following a first-ever stroke and the LDL-C/HDL-C ratio at 3 and 12 months. We found that a high LDL-C/HDL-C ratio protected against death, recurrence, and moderate disability within 3 months following stroke onset. At the 3- and 12-month observation points, the mortality risk in group 2 was 0.45 and 0.58 times lower than that in the lowest-LDL-C/HDL-C ratio group. However, there were no significant associations between recurrence or moderate disability and the LDL-C/HDL-C ratio within 12 months following stroke onset.

Although many studies have confirmed the importance of the LDL-C/HDL-C ratio for predicting the risk of cardiovascular disease (9, 10), there have been no clear conclusions about its relationship with stroke prognosis. The Helsinki Heart Study found that the LDL-C/HDL-C ratio was a strong predictor of recurrence of coronary heart disease among participants with high triglyceride levels over 5 years of follow-up (18). Another previous study that compared patients with normal and abnormal lipid profiles confirmed the ability of the LDL-C/HDL-C ratio to predict future strokes (19). Another cross-sectional study stated that a higher cholesterol ratio was associated with an increased risk of ischemic stroke in Chinese patients with hypertension (20). A 4.9-year follow-up study reported that a two-fold increase in the baseline LDL-C/HDL-C ratio increased the risk of ischemic stroke recurrence by 31% (21).

In contrast to the above results, we found that the LDL-C/HDL-C ratio was inversely associated with the incidence of adverse events (including death, recurrence, and moderate disability) at 3 months after stroke. The risk of death at 12 months in group 2 (i.e., with an LDL-C/HDL-C ratio of 2.23–2.88) was 43% lower than that in group 1 (i.e., with an LDL-C/HDL-C ratio of <2.23). Low HDL-C concentrations at 3 months following stroke protected patients against recurrence of ischemic stroke and moderate disability. However, there was no association between the LDL-C/HDL-C ratio and recurrence or moderate disability at 12 months. Moreover, in the analysis by cerebral infarction subtype, the prognosis following LA was similar to that following ischemic stroke, but the relationship of prognoses and LDL-C/HDL-C ratio are not statistically significant for cardioembolism and SA. This result demonstrates that a high LDL-C/HDL-C ratio is a positive short-term prognostic factor, in terms of death, recurrence, and moderate disability.

The exact mechanisms underlying these relationships remain to be elucidated. Most of the patients in this population had not previously taken lipid-lowering therapy. This phenomenon may explain their higher LDL-C/HDL-C ratio at baseline. Additionally, we treated all enrolled patients with statins. However, while patients may comply with physicians' instructions for 3 months after stroke, most do not strictly adhere to long-term statin use. This tendency may explain the discrepancies between our findings and those of previous studies (21). In this study, the LDL-C/HDL-C ratio was more representative because it accounts for the influence of LDL-C and HDL-C on prognosis.

Previous studies have reported that women tend to be older than men at the onset of stroke (22–24). Consistent with those previous studies, women were older than men at the age of stroke onset in this study. The neuroprotective effects of estradiol in women may explain the older stroke onset age in women (25, 26).

Several limitations to our study need to be addressed. First, our results are based on a patient population recruited from a single center and should be validated in larger populations. Second, we did not obtain information on lipid-lowering therapy before stroke onset and after stroke. However, most patients did not understand whether they had had hyperlipidemia in the past, so they generally had not taken lipid-lowering treatment before stroke onset. This may impact the evaluation of the relationship between stroke outcomes and the LDL-C/HDL-C ratio. Third, to further evaluate the association between lipid parameters and ischemic stroke outcomes, the LDL-C/HDL-C ratio and patients' medication status during follow-up should also have been measured. However, we only analyzed the association between lipid parameters during hospitalization (but not during follow-up) and ischemic stroke outcomes. Fourth, all measurements were performed within the first 72 h after stroke onset; the relatively long interval from stroke onset to lipid measurement may have affected the evaluation of the association between the LDL-C/HDL-C ratio and stroke outcomes. Finally, the follow-up duration should have been longer to enable us to explore the relationship between the LDL-C/HDL-C ratio and long-term prognosis after stroke.

## Conclusion

In contrast with previous studies, we found that an increased LDL-C/HDL-C ratio protected against negative outcomes within 3 months following stroke. However, there was no significant association between stroke recurrence and the LDL-C/HDL-C ratio at 12 months. Our study revealed a positive association between higher LDL-C/HDL-C ratio and short-term prognosis (death, recurrence, and moderate disability) following stroke, emphasizing the value of the LDL-C/HDL-C RATIO for assessing short-term outcomes. These results raise the question of whether it is necessary to minimize the cholesterol ratio as quickly as possible in China.

## Data Availability Statement

The datasets generated for this study are available on request to the corresponding author.

## Ethics Statement

The study was approved by Medical Research Ethics Committee of Heilongjiang Provincial Hospital, and written informed consent was obtained from each participant during the recruitment process.

## Author Contributions

YW contributed to the study design, performed data collection, data interpretation, and critical review. LL performed data analysis and contributed to drafting of the article. LL, PY, CL, JL, ZZ, YL, and SL performed data collection, case diagnoses, and confirmation of case diagnoses. All authors read, revised, and approved the final version of the paper.

## Conflict of Interest

The authors declare that the research was conducted in the absence of any commercial or financial relationships that could be construed as a potential conflict of interest.
